# GABA Supplementation Negatively Affects Cognitive Flexibility Independent of Tyrosine

**DOI:** 10.3390/jcm10091807

**Published:** 2021-04-21

**Authors:** Lee Wei Lim, Luca Aquili

**Affiliations:** 1 Neuromodulation Laboratory, Li Ka Shing Faculty of Medicine, School of Biomedical Sciences, The University of Hong Kong, Hong Kong, China; limlw@hku.hk; 2 College of Health & Human Sciences, Charles Darwin University, Darwin 0810, Australia

**Keywords:** tyrosine, GABA, dopamine, reinforcement, cognitive flexibility

## Abstract

Increasing evidence, particularly from animal studies, suggests that dopamine and GABA are important modulators of cognitive flexibility. In humans, increasing dopamine synthesis through its precursor tyrosine has been shown to result in performance improvements, but few studies have reported the effects of GABA supplementation in healthy participants. We conducted a double-blind, placebo-controlled, randomized experiment to test the interactive effects of tyrosine and GABA administration on two measures of cognitive flexibility, response inhibition and task switching. A total of 48 healthy volunteers were split into four groups (placebo, tyrosine alone, GABA alone, and tyrosine and GABA combined). They completed cognitive flexibility tasks at baseline and after drug administration. We found that tyrosine alone had no impact on the measures of cognitive flexibility, whereas GABA alone and in combination with tyrosine worsened task switching. Our results provide preliminary evidence that putative increases in GABA and dopamine synthesis do not interact to affect cognitive flexibility performance.

## 1. Introduction

Cognitive flexibility is an important executive function that is characterised by the ability to make adaptive responses to changes in stimulus-reward contingencies. Impairments in cognitive flexibility have been reported in psychiatric and neurological disorders including OCD [1], schizophrenia [2], depression [3], eating disorders [4], addiction [5], gambling disorders [6] and Parkinson’s disease [7], and have been extensively reviewed elsewhere [7,8,9,10,11]. Numerous studies in both animals and humans have identified the dopaminergic system (DA) as an important regulator of this function [9]. The administration of the precursor tyrosine is a relatively safe pharmacological approach for manipulating DA (and catecholamine) synthesis. Tyrosine is naturally found in food products and is readily available as a food supplement. Tyrosine loading (supplementation) and tyrosine depletion studies respectively showed improvement and impairment of aspects of cognitive flexibility such as task switching, response inhibition, and reversal learning, [12,13,14,15,16,17,18,19], but some null findings were also reported [20,21,22]. Although the relationship between the inhibitory neurotransmitter GABA and cognitive flexibility is less clear, ventral tegmental area (VTA) GABA neurons have been shown to modulate VTA DA neuron activity in reinforcement-related tasks [23]. In humans, the administration of the GABA_b_ receptor agonist baclofen was shown to affect reinforcement learning in a dose-dependent manner [24,25], as well as reduce drug cravings [26]. Critically, these effects were related to the reduced activation of striatal and prefrontal regions involved in reward processing [27], and the suppression of LTP activity [28]. Several recent studies have suggested that the administration GABA in the form of a food supplement could reduce physiological indicators of stress [29,30,31], and could positively affect aspects of cognition including action selection and temporal visual attention [32,33]. 

Given that the assessment of reward-related information is a crucial element of tasks used to measure cognitive flexibility and based on the baclofen literature, we hypothesize that GABA supplementation may similarly impact the execution of cognitive flexibility tasks. The exact effect of GABA supplementation alone and in combination with tyrosine on cognitive flexibility may be difficult to predict. Part of the difficulty in determining these effects lies in the well-known inverted U-shaped relationship between DA concentration and executive function [34], as well as a similar but more complex relationship suggested for GABA [35]. The authors hypothesized that depending on which cognitive/behavioural process would be examined, GABA neuronal activity may present the following effects: (A) an inverted U-shaped effect, (B) increased neuronal activity above a certain threshold negatively affecting cognition/behaviour, (C) neuronal activity below a certain threshold negatively affecting cognition/behaviour, or (D) a monotonic positive relationship between neuronal activity and cognition/behaviour. 

Nevertheless, our aim was to provide preliminary evidence of a potential interaction between GABA and tyrosine as modulators of cognitive flexibility. To achieve this aim, we recruited 48 healthy volunteers who were administered either tyrosine or GABA alone, a combination of tyrosine and GABA, or a placebo followed by a response inhibition task and a task switching task (two subcomponents of cognitive flexibility). 

## 2. Methods

### 2.1. Participants

This study was approved by the ethics committee of Sheffield Hallam University and was conducted in compliance with the Declaration of Helsinki. Participants were recruited through convenience sampling and consisted of 48 university students (M = 22.8 years, SD = 2.8) including 32 females and 16 males. Notably, the majority of the participants were in the younger age range, which may limit the generalizability of the results to the older population. To the best of our knowledge, there is insufficient evidence to suggest that tyrosine (administered as a single dose) produces differential effects on the cognitive performance of younger versus older healthy participants. A study looking at the effects of habitual daily tyrosine intake (i.e., looking at the long-term effects of tyrosine) on cognition in younger and older adults demonstrated that both groups responded in a similar way to higher daily intake of tyrosine (i.e., both groups reported benefits in measures of fluid intelligence, working memory, and episodic memory) [36]. Written informed consent was obtained from all participants before the start of the study. Participants did not receive any financial compensation. Exclusion criteria were similar to previous studies from our research group [12,15,22,37]. Participants were excluded from the study if they had any cardiac, hepatic, renal, and neurological disorders; A history of alcohol/drug addiction; psychiatric illness; or a history of taking tyrosine or GABA supplements. We did not screen for IQ, which may potentially affect cognitive flexibility performance [38], as our sample consisted entirely of university students who would have entered their respective programs with a similar level of academic achievement, and thus IQ would not likely bias the results, particularly as participants were randomly allocated to the different drug treatments.

### 2.2. Drug Administration

Participants were randomly assigned to four different groups: (1) tyrosine alone, (2) GABA alone, (3) combination of tyrosine and GABA, or (4) placebo. The tyrosine group (*n* = 12) received 2.0 g of tyrosine (BulkPowders Ltd., Colchester, UK), the GABA group (*n* = 12) received 800 mg of synthetic GABA (NutraVita Ltd, Maidenhead, UK) according to a previous protocol [33], the combination group (*n* = 12) received both tyrosine and GABA, and the placebo group (*n* = 12) received 2.0 g of microcrystalline cellulose (Redwells Creative Limited, London, UK). All tyrosine/GABA/cellulose powders were dissolved in 400 mL of orange juice. Following testing, data from 4/48 participants were discarded due to extreme values (Z-scores = ±4) in the pre-drug and/or post-drug cognitive flexibility measurements, resulting in 11 participants in the tyrosine group, 12 in the GABA group, 10 in the tyrosine + GABA group, and 11 in the placebo group. The drugs were administered as a single dose in one session, in line with the vast majority of studies looking at the effects of short-term tyrosine on healthy populations reviewed elsewhere [39].

## 3. Cognitive Flexibility Tasks

**Figure 1:**
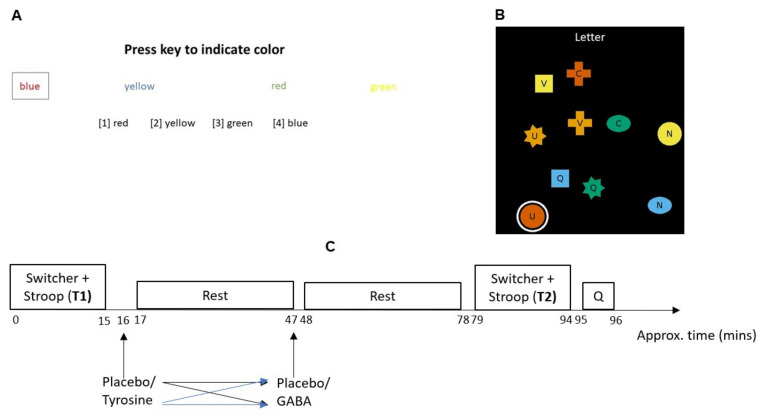
(**A**) Schematic illustration of the Victoria Stroop task and (**B**) switcher task. (**C**) Outline of the experimental procedure.

Response inhibition was assessed by the Victoria Stroop task developed by Strauss and Spreen [40] and task switching was assessed by the Switcher task developed by Mueller [41]. These two cognitive flexibility tests were conducted using PEBL software [42]. The Victoria Stroop task was divided into three blocks, each containing 24 trials. The trials were self-paced, but participants were instructed to respond as quickly as possible. Participants were asked to indicate the colour of individual dots in the first block, whereas in the second block, they indicated the colour of individual words. Both blocks acted as a control for the task. The third block was identical to the second block, except the name of a colour was printed in an ink colour that did not match the name (e.g., yellow was written in green ink; see Figure 1A). Therefore, the third block provides a measure of response inhibition, in which participants must refrain from making an automatic reading response and instead make a colour-naming response. The two primary measures of interest in this task were the number of intrusions or errors that occurred during the third block and the efficiency score, which was calculated by dividing the time taken to complete the third block versus the second block (i.e., on average, participants took longer to complete block three than block two). Normative data and a discussion of the psychometric benefits of this task have been reported elsewhere [43]. The switcher task shares many similarities with the Wisconsin Card Sorting Task (WCST). Notably, it offered an added benefit in our study sample predominantly made up of psychology students, in that participants were less likely to have had any prior experience with this task compared to the WCST, thus eliminating any potential practice effects. The switcher task was divided into nine blocks, each containing six trials. The trials were self-paced, but participants were instructed to respond as quickly as possible. The objective of this task was to measure their ability to flexibly switch between decision rules. Participants were required to select the next stimulus based on two rules that alternated (block 1 to 3), on three rules that alternated but in a consistent order (block 4 to 6), or on three rules that alternated in a random order (block 7 to 9). As in the WCST task, these rules relate to matching the next stimulus based on its shape, letter, or colour. For example, in block 7 to 9, participants may have been asked to select the next stimulus based on a letter rule (trial 1), then a shape rule (trial 2) and a colour rule (trial 3). In trial 4–6, the rules may be based on shape, then colour, and finally letters (see Figure 1B). The two primary measures of interest were the time taken to complete the task and the number of errors.

## 4. Procedures

After screening for eligibility, participants were asked not to eat or drink for a minimum of 3 h prior to testing to reduce competition from other amino acids that share the same transporter [44], as demonstrated in previously published papers by our group. Participants were then required to attend a session lasting approximately 100 min. They first completed the two cognitive flexibility tasks. The order of task presentation (Switcher test and Victoria Stroop test) was randomized across participants. Participants then received either tyrosine or placebo according to the group allocation. After 30 min, they received either placebo or GABA according to the group allocation. At approximately 60 min, participants then completed the two cognitive flexibility tasks for a second time. 

There were two important reasons for administering the drugs in this particular sequence and timing. Tyrosine and GABA administration produce peak plasma concentrations at two different times. For tyrosine, the peak plasma concentration occurs at 60 min [39], whereas for GABA this happens at 30 min [32]. Thus, the tyrosine group first completed the cognitive flexibility tasks (T1, Figure 1C), then immediately received tyrosine, and were retested at 60 min post-drug administration (T2). The GABA group first completed the cognitive flexibility tasks, then received GABA after 30 min, and were retested at 60 min post-drug administration. The tyrosine + GABA group first completed the cognitive flexibility tasks, and then were immediately given tyrosine and after 30 min were given GABA before retesting at 60 min. The placebo group first completed the cognitive flexibility tasks, and then were immediately given placebo and retested at 60 min. To ensure double-blinding, participants in the tyrosine group received tyrosine immediately and a placebo (microcrystalline cellulose) after 30 min; the GABA group received a placebo immediately and GABA after 30 min; whereas the placebo group received microcrystalline cellulose immediately and again after 30 min. Finally, to ensure double blinding had been achieved, participants were asked to fill out a questionnaire in which they were asked to state which condition they had been allocated ((1): placebo, (2): GABA; (3): tyrosine, or (4): tyrosine + GABA), which was followed by a debriefing. A schematic representation of the experimental procedure is shown in Figure 1C. 

## 5. Data Analysis

Statistical analyses were performed using SPSS version 26 (IBM, New York, NY, USA). Sample size was determined using power calculations based on previous findings from our group [15] with the following parameters: a minimum power of 0.8, an alpha level of 0.05, a large effect size (η^2^) of 0.20 based on repeated measures ANOVA, and within-between interaction with four groups and two measurements (G *Power 3.1.9.2, Düsseldorf, Germany). All cognitive flexibility measures of interest were analysed using a 4 × (2) mixed factorial ANOVA with the drug as the between subject factor (placebo, GABA, tyrosine, and tyrosine + GABA) and time as the within subject factor (time 1 (pre-drug) and time 2 (post-drug)). The double-blind efficacy of drug allocation was analysed using a chi-square test.

## 6. Results

### 6.1. Switcher Task

**Figure 2:**
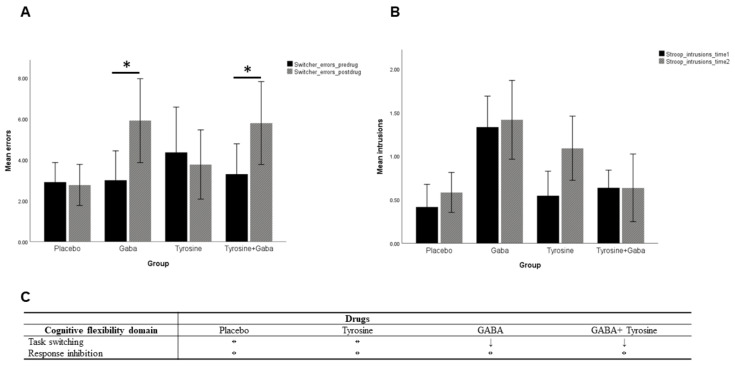
(**A**). Switcher task errors. The clustered bar chart shows a significant time x drug interaction effect. Simple main effects analyses demonstrated errors were significantly increased from baseline to post-drug for the GABA group and tyrosine + GABA group, but not for the placebo group and tyrosine group. (**B**). Victoria Stroop task intrusions/errors. There were no significant interaction effects between time and drugs. (**C**). Schematic summary of the main findings. * indicates *p* ≤ 0.05. ↔ indicates unchanged performance. ↓ indicates significant worsening of performance.

The first measure of interest investigated in the switcher task was the number of errors. There was a significant main effect of time (F (1, 40) = 5.12, *p* = 0.029, η^2^ = 0.11) with errors increasing from pre-drug (time 1) to post-drug (time 2), but no main effect of drugs (F (3, 40) = 0.14, *p* = 0.866, η^2^ = 0.01). Importantly, there was a significant interaction effect between time and drugs (F (3, 40) = 3.04, *p* = 0.040, η^2^ = 0.18). To break down this interaction, follow-up simple main effect analyses were performed. For the placebo group, there was no significant difference in the errors between pre-drug and post-drug (F (1, 40) = 0.02, *p* = 0.896), which was also the same for the tyrosine group (F (1, 40) = 0.33, *p* = 0.571). However, for those participants taking GABA either alone (F (1, 40) = 8.61, *p* = 0.005, η^2^ = 0.17) or in combination with tyrosine (F (1, 40) = 5.32, *p* = 0.026, η^2^ = 0.11), the errors increased from pre-drug to post-drug, indicating a task switching impairment post-drug (See Figure 2A). There were no significant differences when comparing the four different groups at time 1 (*p =* 0.911) or at time 2 (*p =* 0.510).

The second measure of interest investigated in the switcher task was the time taken to complete the task. There were neither significant main effects (*p* ≥ 0.231) nor a significant interaction effects (*p =* 0.530) across groups.

### 6.2. Victoria Stroop Task

The two measures of interest in the Victoria Stroop task were the efficiency score and the number of errors/intrusions. There were neither significant main effects (*p* ≥ 0.318) nor a significant interaction effects (*p =* 0.791) in the efficiency score. Similarly, there were neither significant main effects (*p* ≥ 0.074) nor a significant interaction effects (*p =* 0.872) for errors/intrusions (See Figure 2A,B).

A summary of the results for both the switcher and Victoria Stroop tasks is given in Figure 2C.

### 6.3. Double Blinding of the Drug Administration

The double-blinding efficacy of drug administration was analysed using a chi-square test. Because one of the assumptions of the chi-square test is that no expected value should be lower than 5, we analysed these data collapsing the scores for placebo and tyrosine into one group (*n* = 22), and GABA and GABA + tyrosine into another group (*n* = 22). There was no significant association between the actual condition and the condition correctly identified by the participant (χ^2^ (1) = 0.49, *p* = 0.736). 

## 7. Discussion

There are three main takeaway messages from the current study findings: (1) GABA supplementation negatively affected only the task switching aspect of cognitive flexibility, but not response inhibition, (2) this effect also occurred when tyrosine was co-administered, and (3) tyrosine did not affect any measures of cognitive flexibility. In discussing the current findings, we will largely focus on previous investigations that (A) were based on healthy participants, (B) measured cognitive flexibility, (C) used tyrosine, or (D) administered a GABA supplement and/or GABA agonist.

A parsimonious behavioural interpretation of the two latter findings is that tyrosine alone did not significantly alter performance, and thus its administration in conjunction with GABA was not sufficient to rescue the impairment caused by GABA alone. The null effects of tyrosine supplementation on cognitive flexibility are at odds with previous studies in humans [13,17], including some of the findings from own research group [15]. Notably, in the latter study, while reversal learning (a subdomain of cognitive flexibility) was improved following tyrosine administration, task switching did not, as measured by the WCST. Moreover, we reported a negative effect of tyrosine administration on the WCST under cognitively demanding conditions [22]. Given the similarities between the Switcher task and the WCST, we could tentatively conclude that tyrosine is not involved in task switching. Nevertheless, in a follow-up study by our research group, in which dopamine levels were putatively reduced by an acute phenylalanine/tyrosine depletion procedure (APTD), reversal learning remained unaffected, while task-switching performance was impaired [12].

Taken together, these findings point to a complex relationship between tyrosine supplementation/depletion and cognitive flexibility. Some of these complexities may also relate to sub-domains of cognitive flexibility (response inhibition, reversal learning and task switching) that are under the control of a differential neuronal network [5,45].

In regard to GABA’s effects, it is worth noting that several studies in animals have suggested that GABA may not be able to enter the blood brain barrier (BBB) [46,47]. This point, if true, may be particularly relevant, as it would suggest that the numerous reports by individuals consuming GABA supplements may have been driven by a placebo effect [48]. However, some recent evidence has demonstrated that GABA can in fact enter the BBB, although the reported concentrations were small [49,50]. Furthermore, in a study by Takanaga et al. [51], a GABA transporter was found in the BBB, indicating that it is possible for GABA to move in and out of the brain. Ultimately, however, there have been no magnetic resonance spectroscopy (MRS) investigations carried out in humans that have revealed the effects of GABA administration on GABA concentrations, and such studies will be needed to clarify this issue. Nevertheless, the reported physiological and cognitive effects in the literature (including the results from this report) point to GABA administration having a modulating effect, albeit the mechanisms of this remain to be elucidated.

Only two studies have investigated the use of GABA as a supplement in humans, which reported a beneficial effect on two different aspects of cognition [32,33]. In our study, we found that GABA administration impaired cognitive flexibility performance. Given the suggested non-linear relationship between GABA concentration and cognitive performance [35], it is plausible that the dosage used in this study caused a negative shift in performance similar to that observed for the U-shaped relationship between DA and cognition. Again, the use of MRS could prove to be an invaluable tool to answer this question.

Very little evidence is available with respect to the acute administration of GABA_A_/_B_ agonists/antagonists and its effects on measures of cognitive flexibility in healthy participants. Administration of the GABA_A_ receptor agonist, Diazepam, in healthy participants has been shown to impair response inhibition [52]; however, other investigations have found this effect to be dose-dependent, with therapeutic doses (e.g., 5–10 mg) having no impact [53], and larger doses (20 mg) disrupting response inhibition performance [54].

Based on the literature previously reviewing the use of baclofen (a GABA_B_ receptor agonist) in humans, it is possible, albeit speculative (i.e., relying on data from animal studies), to suggest that GABA supplementation acts to inhibit a DA reward signal (VTA-striatum-prefrontal cortex) that is essential for learning. This idea was corroborated by optogenetic manipulations of VTA GABA neurons, in which it was shown that activating these neurons could disrupt reward consumption [55]. Importantly, VTA GABA neurons provide information to VTA DA neurons about how much reward to expect. Crucially, they have been demonstrated to have a causal role in reward prediction error calculations performed by DA cells [56]. These reward prediction error calculations, if disrupted, would in turn negatively affect task switching performance, as demonstrated by the switcher task, in which stimulus-reward contingencies were unexpectedly and unpredictably changed. Furthermore, impairments in reward prediction error have been reported in psychiatric and neurological disorders including depression [57], schizophrenia [58] and addiction [59], highlighting links between GABA and DA function, cognitive flexibility, reward prediction error and disease.

Finally, a potential limitation of the current study is that it was powered to only detect a statistically significant result with a large effect size (η^2^ = 0.18). The rationale behind choosing the sample size was based on previous findings from our research group [15] using a similar methodological approach.

In conclusion, we provide preliminary evidence that GABA supplementation in humans negatively impacts cognitive flexibility and this occurred irrespective of the putative increase in DA synthesis.

## Data Availability

Data available upon request.
